# Comprehensive analysis of the role of deep inspiration breath‐hold in right‐sided breast cancer radiotherapy: A focus on cardiac substructures and right coronary artery

**DOI:** 10.1002/acm2.70216

**Published:** 2025-08-21

**Authors:** Volkan Semiz, Barbaros Aydin, Dilara Gulsan, Ece Atac, Ece Ozkaya, Seyda Kinay, Dogukan Akcay, Recep Kandemir, Melek Can, Ilknur Gorken

**Affiliations:** ^1^ Department of Radiation Oncology Dokuz Eylul University Faculty of Medicine Izmir Turkey; ^2^ Department of Radiation Oncology Izmir City Hospital Izmir Turkey; ^3^ Vocational School of Health Services Dokuz Eylul University Izmir Turkey

**Keywords:** breast cancer, cardiac substructures, deep inspiration breath hold, dosimetry, radiotherapy, right coronary artery

## Abstract

**Purpose:**

The deep inspiration breath‐hold (DIBH) technique, a method used to minimize radiation exposure to normal tissues in breast cancer patients, has primarily been applied to patients with left breast cancer. However, this study, with its unique focus on right breast cancer, aims to evaluate the potential benefits of DIBH on the right coronary artery (RCA), cardiac substructures, and liver dose in right breast cancer radiotherapy.

**Materials and Methods:**

Forty right‐sided breast cancer patients were included in this study. Radiotherapy planning using 3D, IMRT, and VMAT was performed using both free‐breathing (FB) and DIBH techniques. Two treatment plans were generated per patient, and organ‐at‐risk (OAR) doses were compared using the Wilcoxon signed‐rank test. Patients were analyzed based on regional nodal irradiation (RNI) status.

**Results:**

The mean radiation doses (Dmean) to the liver, ipsilateral lung, heart, and RCA region were 5.4, 15.9, 2.5, and 6 Gy with FB, decreasing to 4.6, 12.6, 1.6, and 3.6 Gy with DIBH. For the left ventricle, right ventricle, left atrium, and right atrium, the Dmean values were 1.2, 2.2, 2, and 4.6 Gy with FB, versus 0.76, 1.8 Gy, 1.2, and 3.2 Gy with DIBH. DIBH significantly reduced liver and lung doses in all patients, while heart and RCA doses reductions were observed only in those receiving RNI.

**Conclusion:**

The use of the DIBH technique can significantly reduce radiation exposure to OARs in right‐sided breast cancer radiotherapy. Despite a slight increase in treatment duration, DIBH should be considered for right breast cancer radiotherapy to minimize radiation‐related toxicity.

## 1 INTRODUCTION

Breast cancer is the most common malignancy among women, and survival rates have significantly improved due to multidisciplinary treatment approaches.^1^ Radiotherapy (RT) plays a crucial role in reducing the risk of recurrence and enhancing long‐term survival by improving local control, both after breast‐conserving surgery and post‐mastectomy.^2^, ^3^

However, while RT offers survival benefits, it also carries a potential risk of toxicity due to radiation exposure to surrounding healthy structures. The doses to organs at risk (OAR) during breast RT vary based on individual anatomy, laterality, irradiated targets, and the techniques used. Particularly in cases of regional nodal irradiation (RNI) for left‐sided breast cancer, radiation exposure to the lungs, heart, and coronary arteries can significantly influence long‐term non‐cancer‐related mortality.^4^, ^5^ Consequently, contemporary RT planning strategies necessitate the integration of organ‐sparing techniques.

Deep inspiration breath‐hold (DIBH) is a technique in which the patient takes a deep breath and holds it during the inspiratory phase, thereby increasing thoracic volume and expanding the anatomical distance between the heart and the chest wall. This spatial displacement effectively reduces radiation exposure to cardiac and coronary arteries, thereby minimizing dose‐related toxicity.^6^ The DIBH technique has been successfully implemented for many years, particularly in patients with left‐sided breast cancer, leading to a decrease in radiation‐induced cardiac mortality.^7^, ^8^, ^9^, ^10^ Although the DIBH technique is generally well tolerated, patient's selection should be carefully considered, taking into account factors such as the patient's ability to comply with the technique, cost, treatment duration, and patient characteristics.

DIBH is presumed to be most beneficial for patients undergoing left‐sided breast irradiation due to the anatomical proximity of the heart and coronary vessels to the radiation field. In case of right breast irradiation, organs such as the liver, heart and lungs may receive a significant radiation dose, potentially leading to long‐term side effects. However, the application of DIBH for right‐sided breast cancer remains limited, with only a few studies investigating its utility in this context. In our study, we aim to evaluate the impact of DIBH on the dose distribution to the heart and its substructures, including the right coronary artery (RCA) and left anterior descending artery (LAD), as well as to the lungs and liver, in patients with right‐sided breast cancer.

## 2 MATERIALS AND METHODS

### 2.1 Patient selection and treatment planning

Patients diagnosed with right breast cancer who underwent RT between 2020 and 2022 and were able to comply with the DIBH technique were included in this study. All patients in this study were treated in the head‐first supine (HFS) position. The cohort included both intact breast and post‐mastectomy patients. Patients were divided into two groups: those who received whole‐breast RT with or without RNI, including internal mammary nodes (IMN). Patients who received lymphatic irradiation without IMN involvement were not included in the study.

All eligible patients received pre‐treatment training on DIBH, conducted by a highly experienced and skilled technician, 1–3 days prior to imaging. Patients who successfully adhered to the technique underwent computed tomography (CT) simulation in free breathing (FB) and DIBH conditions. For appropriate cases, intravenous contrast‐enhanced imaging was performed to facilitate the delineation of coronary arteries. CT (Siemens Somatom Definition AS) scans were acquired with patients positioned supine on a dedicated breast board, arms raised above the head, and scanned with a slice thickness of 3 mm in FB. DIBH scans were performed after the FB scans.

The DIBH technique was implemented using the Real‐Time Position Management (RPM) system (Varian Medical Systems, Palo Alto, CA). The RPM reflective marker block was placed along the midline of the patient's body near the xiphoid process. Before image acquisition, patients were allowed to breathe normally to establish a stable respiratory pattern. Subsequently, patients were instructed to hold their breath for at least 20 s during deep inspiration. The upper and lower gating thresholds were set 2.5 mm above and below the breath‐hold level, resulting in a total gating window of 5 mm. The respiratory cycle was continuously monitored via the RPM system, and CT acquisition was initiated once the patient demonstrated at least three consecutive stable breath‐holds. The scan was performed to encompass the entire liver and surrounding structures as required for treatment planning.

For each patient, the liver, lungs, contralateral breast, spinal cord, esophagus, heart, LAD, RCA, right and left ventricles (RV and LV), and right and left atria (RA and LA) were contoured as OAR in both FB and DIBH CT scans.^11^ Based on the stage of the disease, the decision was made to irradiate whole‐breast or regional lymphatics (level 1–3, supraclavicular area, and IMN) for the patients, and clinical target volumes (CTV) were contoured according to the ESTRO guidelines.^12^ Afterward, planned target volumes (PTV) were generated, and a dose of 50 Gy in 25 fractions was prescribed. For patients who were to receive a boost to the tumor bed, boost plans were created sequentially using a new planning CT scan. Dosimetric evaluation excluded any sequential boost, as not all patients received a sequential boost.

Treatment planning was performed using the Varian Eclipse system (version 15.1) on the DIBH CT scan, with 3D conformal RT (3DCRT), Intensity‐Modulated Radiation Therapy (IMRT), or Volumetric Modulated Arc Therapy (VMAT) techniques applied. Treatment plans were created for each patient using all three methods, and the most appropriate plan was selected. All radiotherapy plans were generated by two full‐time medical physicists, with each patient's pair of plans created by the same individual to ensure internal consistency. Both physicists were supervised by a senior physicist specialized in breast radiotherapy. Although the planners were aware that a retrospective study was being conducted, they were blinded to the study hypothesis. All final plans were reviewed and approved jointly by a senior radiation oncologist and a radiation oncology resident to ensure clinical accuracy and consistency

The same written treatment planning directive was set according to QUANTEC guidelines (Table S1).^13^ Briefly, for the ipsilateral lung, the mean dose (Dmean) was limited to < 18 Gy, with V20 < 30%, and for the heart, the Dmean was limited to < 5 Gy. In cases receiving RNI, the V20 dose for the ipsilateral lung was accepted up to 35%. For the contralateral breast, contralateral lung, liver, LAD, and RCA, the principle of “as low as reasonably achievable” (ALARA) was applied with an additional goal of maintaining the RCA mean dose below 10 Gy, while no dose limits were defined for the cardiac substructures. Treatment plans were accepted if at least 95% of the PTV received 95% of the prescribed dose, the maximum dose (Dmax) was < 107%, and the OAR dose criteria were met. Treatments were delivered using a TrueBeam© (Varian Inc., Palo Alto, CA) Linear Accelerator equipped with a 120‐leaf Millennium Multileaf Collimator with a 0.5 mm leaf width. During treatment, patients were instructed to hold their breath using video and audio support. Daily IGRT was performed by acquiring a Cone Beam CT (CBCT) to verify the patient's position and accurate dose delivery

Treatment plans were created on the FB CT retrospectively using the same RT technique, optimization parameter and same PTV coverage criteria as for DIBH. FB plans were used for dosimetric comparison purposes only.

Treatment duration was calculated from the setup start to treatment completion and obtained from the treatment planning system. To compare of treatment durations, the treatment times of 40 patients who received right breast RT, including 16 treated for the whole‐breast and 24 treated for RNI, were evaluated.

This study was approved by the Ethics Committee of Dokuz Eylul University (No: 2025/9959) and conducted in accordance with the principles of the Declaration of Helsinki

### 2.2 Statistical analysis

For OARs, dose‐volume histograms (DVHs) were used to assess the fractional percentage of volumes receiving x Gy (Vx%) as well as the maximum dose (Dmax) and mean dose (Dmean). These were reported and compared. The Wilcoxon signed‐rank test was used to examine differences in DVH parameters between FB and DIBH plans. Statistical analyses were performed using IBM SPSS Statistics (Version 29.0.2.0, IBM Corp) and GraphPad Prism (version 10.4.1). A two‐tailed *p*‐value < 0.05 was considered statistically significant.

## 3 RESULTS

**FIGURE 1:**
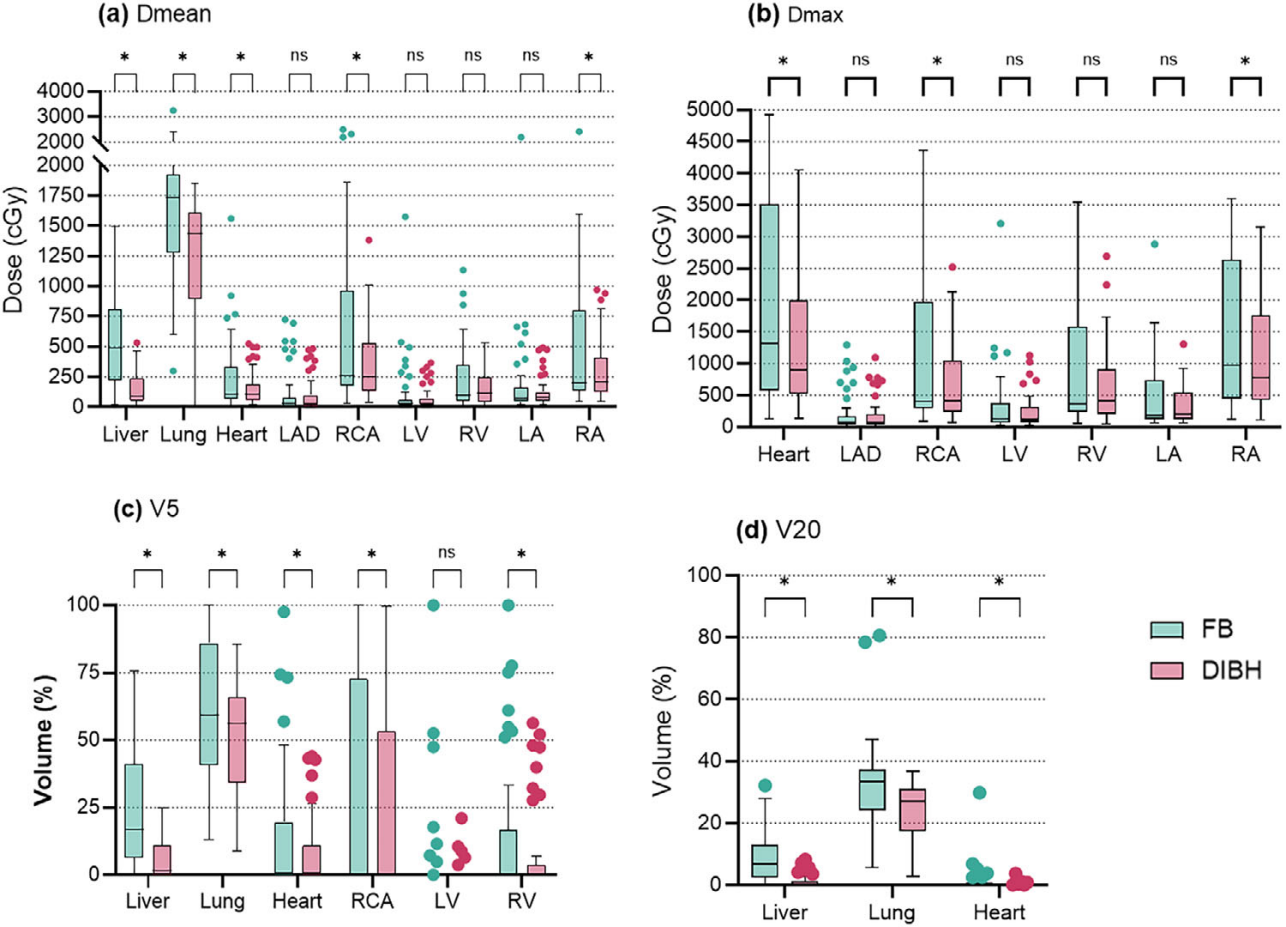
Organ at risk doses. *:*p* < 0.05, ns: not significant. LAD: Left anterior descending artery, RCA: Right coronary artery, LV: Left ventricle, RV: Right ventricle, LA: Left atrium, RA: Right atrium, FB: Free breath, DIBH: Deep inspiration breath hold.

In this study, 40 patients treated between 2020 and 2022 were evaluated. The median age of the patients was 50 years (range 27–71). The mean height was 161.6 ± 6.0 cm, and the mean weight was 64.9 ± 10.1 kg. Whole‐breast irradiation was performed in 16 (40%) patients, while 24 (60%) patients received RNI. The RT technique used was VMAT in 7 (17.5%) patients, IMRT in 25 (62.5%) patients, and field‐in‐field 3DCRT in the remaining 8 (20%) patients (Table 1). During the planning phase, the primary priority was to ensure adequate PTV coverage, and no inadequate PTV coverage was observed in either FB or DIBH plans. Since patients were treated according to the DIBH plans, there were no plans that failed to meet the OAR dose criteria. However, in the FB plans, the mean lung dose in 12 patients, the Lung V20 dose in 18 patients, and the heart Dmean dose in 6 patients were outside the acceptable criteria. This occurred as an effort was made to achieve a similar PTV coverage as in the DIBH plans, which naturally led to higher doses to the surrounding organs at risk in some FB cases. OAR Dmean, Dmax, V5, and V20 dose comparisons for the entire patient cohort are shown in Figure 1. Table 2 shows OAR dose comparisons with respect to the presence of RNI between DIBH and FB.

**TABLE 1:** Radiotherapy techniques.

	3DCRT	IMRT	VMAT
RNI (−)	2	13	1
RNI (+)	6	12	6

Abbreviations: 3DCRT, 3D conformal radiotherapy; IMRT, intensity‐modulated radiation therapy; RNI, regional lymphatic irradiation; VMAT, volumetric modulated arc therapy.

**TABLE 2:** Comparison of OAR doses with respect to the presence of RNI.

	Whole‐breast (*n* = 16)			Breast +RNI (*n* = 24)		
OAR	FB	DIBH	*p* value (Z)^a^	FB	DIBH	*p* value (*Z*)^a^
Liver						
Dmean	352 cGy	84 cGy	**<0,001 (−3.51)**	678 cGy	196 cGy	**<0,001 (−4.28)**
V5	12.4 %	2 %	**<0,001 (−3.41)**	32.3 %	24.9 %	**<0,001 (−4.24)**
V10	9.4 %	1.5 %	**<0,001 (−3.40**	21.5 %	3.9 %	**<0,001 (−4.20)**
V20	5.8 %	0.7 %	**<0,001 (−3.30)**	10.8 %	1.5 %	**<0,001 (−4.20)**
Lung						
Dmean	1123 cGy	919 cGy	**0.003 (−2.94)**	1898 cGy	1498 cGy	**<0,001 (−4.28)**
V5	39.9 %	35.7 %	**0.023 (−2.27)**	73.4 %	64.5 %	**<0,001 (−3.60)**
V20	26.1 %	17.3 %	**0.001 (−3.20)**	36.6 %	29.6 %	**<0,001 (−3.47)**
Heart						
Dmax	1400 cGy	851 cGy	0.179 (−1.34)	2390 cGy	1553 cGy	**0.001 (−3.28)**
Dmean	30 cGy	75 cGy	0.352 (−0.93)	37 cGy	217 cGy	**0.009 (−2.60)**
V5	5 %	1.9 %	0.611 (−0.50)	21 %	16.2 %	**0.014 (−2.45)**
V20	0.5 %	0.1 %	0.715 (−0.36)	2.1 %	0.7 %	**<0.001 (−3.32)**
LAD						
Dmax	95 cGy	82 cGy	0.438 (−0.77)	305 cGy	280 cGy	0.107 (−1.61)
Dmean	54 cGy	37 cGy	0.134 (−1.50)	168 cGy	144 cGy	0.943 (−0.70)
RCA						
Dmax	439 cGy	277 cGy	0.501 (−0.67)	1547 cGy	926 cGy	**0.002 (−3.14)**
Dmean	312 cGy	144 cGy	0.134 (−1.50)	789 cGy	512 cGy	**0.002 (−3.17)**
V5	6.3 %	1.5 %	0.285 (−1.06)	47.4 %	35.2 %	**0.003 (−2.94)**
V10	6.2 %	0 %	0.317 (−1.00)	29.2 %	11.4 %	**0.002 (−3.04)**
Left ventricule						
Dmax	144 cGy	110 cGy	0.501 (−0.67)	474 cGy	339 cGy	0.361 (−0.91)
Dmean	43 cGy	24 cGy	0.088 (−1.70)	175 cGy	110 cGy	0.456 (−0.74)
V5	2.9 %	0 %	0.317 (−1.00)	8.1 %	2.1 %	0.128 (−1.52)
Right ventricule						
Dmax	364 cGy	253 cGy	0.469 (−0.72)	1209 cGy	865 cGy	0.052 (−1.94)
Dmean	107 cGy	62 cGy	0.959 (−0.05)	308 cGy	259 cGy	0.407 (−0.82)
V5	4.8 %	0.5 %	0.655 (−0.44)	20.6 %	14.3 %	**0.036 (**−**2.10)**
V10	2.6 %	0 %	0.317 (−1.00)	5.8 %	0.8 %	**0.037 (**−**2.09)**
Left atrium						
Dmax	199 cGy	186 cGy	0.485 (−0.70)	666 cGy	423 cGy	**0.043 (−2.02)**
Dmean	85 cGy	64 cGy	0.679 (−1.41)	446 cGy	168 cGy	**0.038 (−2.07)**
Right atrium						
Dmax	1059 cGy	745 cGy	**0.039 (−2.06)**	1924 cGy	1334 cGy	**0.013 (−2.31)**
Dmean	240 cGy	175 cGy	0.326 (−0.98)	620 cGy		**0.009 (−2.63)**

*Note*: Bold values represent statistically significant values.

Abbreviations: LAD, left anterior descending artery; RCA, right coronary artery; OAR, organ‐at‐risk; RNI, regional lymphatic irradiation; FB, free breath; DIBH, deep inspiration breath hold.

^a^ Wilcoxon signed rank test.

Patient's average treatment time (setup and irradiation time) was 12.23 ± 5.4 min. During the same period in our clinic, the treatment time for patients treated with FB was 10.5 ± 4.7 min. The treatment time with DIBH was, on average, 16% longer.

### 3.1 Liver

The DIBH technique showed the greatest benefit in reducing liver dose. With DIBH, in 81% of patients, no liver volume received 20 Gy. In the entire patient cohort, the mean liver doses (Dmean, V5, V10, and V20) with FB were 5.47 ± 0.6 Gy, 24.3 ± 3.4%, 17.7 ± 2.3%, and 8.7 ± 1.2%, respectively. With DIBH, these values were reduced to 1.51 ± 0.2 Gy, 5.2 ± 1%, 2.9 ± 0.7%, and 1.1 ± 0.3%, respectively.

In whole‐breast irradiation patients, Dmean decreased by 67% (from 352 ± 261 cGy to 84 ± 88 cGy, *Z* = −3.51, *p* < 0.001). V5, V10, and V20 also significantly decreased by 85% (*Z* = −3.41, *p* < 0.001), 86% (*Z* = −3.40, *p* < 0.001), and 86% (*Z* = −3.30, *p* < 0.001), respectively.

For RNI patients, Dmean dropped by 64% (from 678 ± 410 cGy to 196 ± 141 cGy, *Z* = −4.28, *p* < 0.001), with V5, V10, and V20 reduced by 73% (*Z* = −4.24, *p* < 0.001), 80% (*Z* = −4.20, *p* < 0.001), and 92% (*Z* = −4.20, *p* < 0.001), respectively.

### 3.2 Ipsilateral lung

**FIGURE 2:**
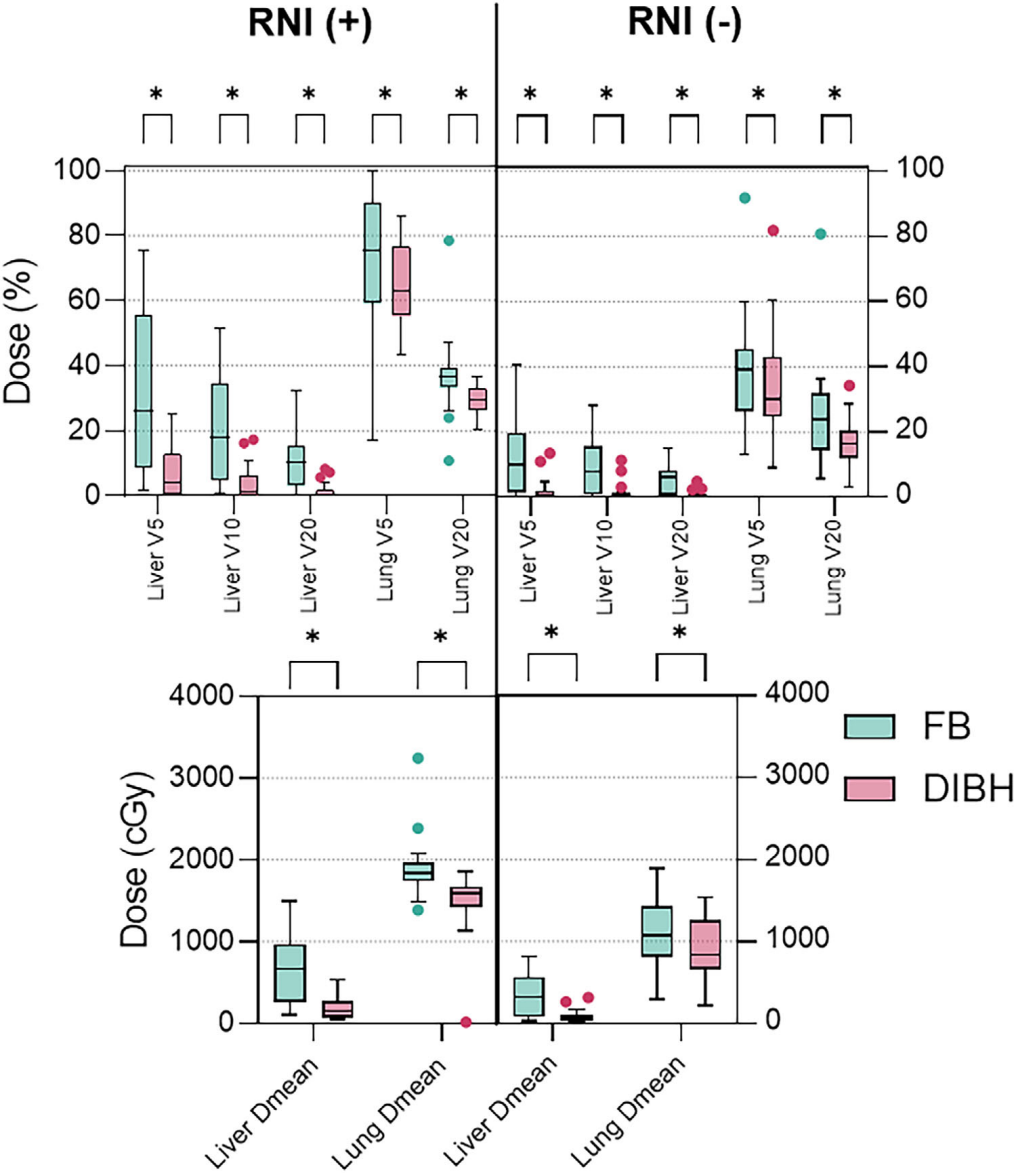
Liver and Lung doses. *:*p* < 0.05 FB: Free breath, DIBH: Deep inspiration breath hold, RNI: regional lymphatic irradiation.

Liver and lung doses are shown in Figure 2. DIBH compared to FB showed a significant decrease in lung Dmean, V5, and V20 doses, regardless of RNI. In the entire patient cohort, the lung doses (Dmean, V5, and V20) with FB were 15.8 ± 0.8 Gy, 60 ± 4%, and 32.3 ± 2.3%, respectively. With DIBH, these values were reduced to 12.6 ± 0.7 Gy, 53 ± 3.2%, and 24.7 ± 1.3%, respectively

In whole‐breast RT patients, Dmean decreased by 27% (from 1123 ± 425 cGy to 919 ± 370 cGy, *Z* = −2.94, *p* = 0.003). V5 and V20 dropped by 10% (*Z* = −2.27, *p* = 0.023) and 27% (*Z* = −3.20, *p* = 0.001), respectively. Dose parameters remained unchanged in three patients, while one showed an increase with DIBH.

In RNI patients, Dmean decreased by 21% (from 1898 ± 354 cGy to 1498 ± 356 cGy, *Z* = −4.28, *p* < 0.001). V5 and V20 dropped by 12% (*Z* = −3.60, *p* < 0.001) and 19% (*Z* = −3.47, *p* < 0.001), respectively. Despite a larger irradiated lung volume, the impact of volume increase was more limited but statistically more significant than in whole‐breast RT patients. In three cases, some dose parameters remained unchanged or increased.

### 3.3 LAD and RCA

**FIGURE 3:**
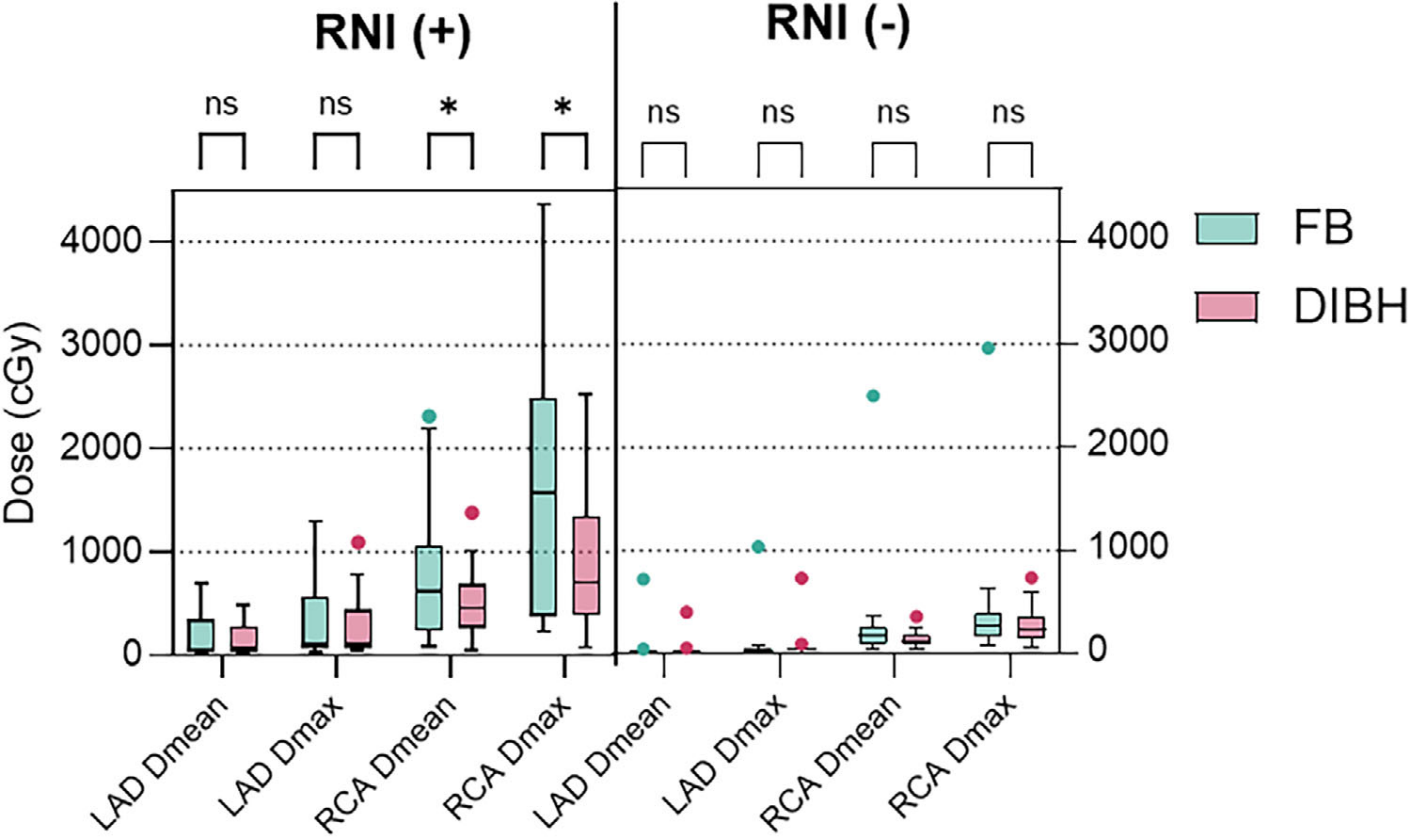
LAD and RCA doses *:*p* < 0.05, ns: not significant FB: Free breath, DIBH: Deep inspiration breath hold, LAD: Left anterior descending artery, RCA: Right coronary artery, RNI: regional lymphatic irradiation.

LAD and RCA doses are shown in Figure 3. Unlike left‐sided treatment, the LAD received the lowest dose in the right breast RT. In the entire patient cohort, the Dmean doses of LAD and RCA with FB were 1.2 ± 0.3 Gy and 6 ± 1 Gy. With DIBH, these values were reduced to 1 ± 0.2 Gy, and 3.6 ± 0.4 Gy.

In whole‐breast irradiation, no significant reductions were observed in Dmean (from 54 ± 178 cGy to 37 ± 98 cGy, *Z* = −1.5, *p* = 0.134) or Dmax (*Z* = −0.77, *p* = 0.438). Similarly, in RNI patients, no significant decrease was found (Dmean: *Z* = −0.70, *p* = 0.943; Dmax: *Z* = −1.61, *p* = 0.107).

No significant reductions were observed for RCA in whole‐breast RT (*p* values for Dmean, Dmax, V5, V10 all > 0.05). However, with DIBH, no patients received more than 10 Gy, compared to one patient in FB.

In RNI patients, significant reductions were seen: Dmean of RCA decreased by 15% (from 789 ± 650 cGy to 512 ± 312 cGy, *Z* = −3.17, *p* = 0.002). Dmax, V5, and V10 also significantly decreased (*Z* = −3.14, *p* = 0.002; *Z* = −2.94, *p* = 0.003; *Z* = −3.04, *p* = 0.002). With DIBH, V10 for RCA was 0% in all but five patients, highlighting IMN irradiation as the key factor in reducing RCA doses.

### 3.4 Heart and substructures

**FIGURE 4:**
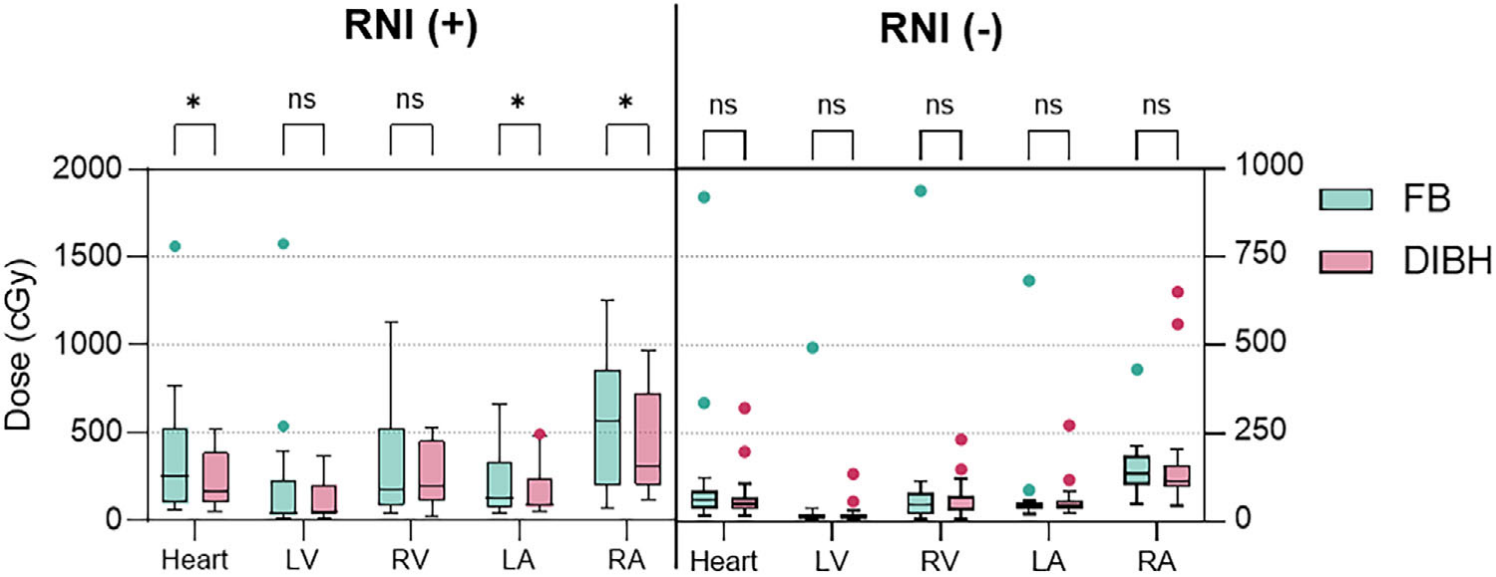
Dmean doses of the heart and subunits *:*p* < 0.05, ns: not significant FB: Free breath, DIBH: Deep inspiration breath hold, LV: Left ventricle, RV: Right ventricle, LA: Left atrium, RA: Right atrium.

Dmean doses of the heart and subunits are shown in Figure 4. In our study, the heart and its substructures were evaluated separately. In FB plans, six patients had heart Dmean doses exceeding acceptable limits, all of whom received IMN irradiation. In the entire patient cohort, the mean heart doses (Dmean, V5, and V20) with FB were 2.5 ± 0.5 Gy, 14.6 ± 3.9%, and 1.4 ± 0.7%, respectively. With DIBH, these values were reduced to 1.6 ± 0.2 Gy, 8.4 ± 2.2 %, and 0.2 ± 0.1%, respectively. For the left ventricle, right ventricle, left atrium, and right atrium, the Dmean values were 1.2 ± 0.4, 2.2 ± 0.4, 2 ± 0.6, and 4.6 ± 0.8 Gy with FB, versus 0.76 ± 0.1, 1.8 ± 0.3, 1.2 ± 0.2, and 3.2 ± 0.4 Gy with DIBH.

In whole‐breast RT, DIBH showed no significant benefit for heart and substructure doses (Dmean decreased from 130 ± 223 cGy to 75 ± 78 cGy, *Z* = −0.90, *p* = 0.179). Only the RA Dmax showed a significant reduction (*Z* = −2.06, *p* = 0.039).

In RNI patients, DIBH significantly reduced heart Dmean (from 337 ± 343 cGy to 217 ± 155 cGy, *Z* = −2.60, *p* = 0.009), with the most notable improvement in V20 (*Z* = −3.32, *p* < 0.001). V20 remained below 1% in all but one patient. While LV and RV Dmean and Dmax showed no significant changes, RV V5, V10, and doses in both atria significantly decreased (Table 2).

## 4 DISCUSSION

The DIBH technique is widely employed in left breast cancer RT due to its potential to reduce radiation doses to the heart and the LAD. However, there are only a limited number of studies evaluating its use in right breast radiotherapy, most of which are dosimetric analyses involving small patient groups and typically focus on a single organ at risk (OAR).^14^, ^15^, ^16^, ^17^, ^18^ A recent meta‐analysis demonstrated the benefits of DIBH in the right breast RT.^19^ In our study, we evaluated multiple OARs simultaneously, including cardiac subunits, in 40 patients who underwent either whole‐breast irradiation or regional nodal irradiation (RNI). Additionally, we assessed treatment duration with DIBH to better understand its potential impact on routine clinical workflow.

Breast RT is associated with an increased risk of various cardiac complications, including coronary artery disease (CAD), cardiomyopathy, pericardial diseases, valvular disorders, and arrhythmias.^5^, ^20^ Minimizing unnecessary radiation exposure to the heart is essential to mitigate these risks. Recent studies indicate that modern RT techniques significantly reduce the risk of cardiac mortality.^21^, ^22^ However, cardiotoxic treatments such as anthracycline‐based chemotherapy and trastuzumab continue to pose a considerable risk of cardiac morbidity. While DIBH is predominantly used for left breast RT, reducing cardiac doses in right breast RT is also important, particularly for patients with pre‐existing heart conditions.^23^ Even in patients receiving RT to the breast alone, the incidence of ischemic heart disease increases. In the study by Darby et al., it was demonstrated that for every 1 Gy increase in mean heart dose, the risk of major coronary events rises by 7.4%, independently of pre‐existing heart disease.^5^ This risk rises in the fifth year post‐RT and persists for up to three decades.

In our study, DIBH significantly reduced the heart dose. For patients undergoing RNI, all cardiac dosimetric parameters showed substantial reductions, specifically, the mean heart dose decreased from 3.3 to 2.1 Gy. However, in patients receiving RT to the breast alone, DIBH did not demonstrate a significant contribution. The reduction in heart doses observed in RNI patients can be attributed to the movement of the IM nodes away from the heart during inspiration. Similar findings were reported by Lai et al.,^17^ although Pandelli^24^ and Esser^15^ did not observe this benefit. A recent meta‐analysis has shown that DIBH effectively reduces both mean heart dose and V5 dose.

When considering cardiotoxicity, the atria and ventricles should also be taken into account.^25^, ^26^ Van den Bogaard et al. highlighted that the left ventricular V5 dose is a stronger predictor of cardiac events than the mean heart dose.^27^ Consistent with this, our study assessed the mean and V5 doses for both the ventricles and atria. The right atrium received the highest radiation, followed by the right ventricle. In RNI patients, DIBH reduced the right ventricular V5 and V10 doses, although the Dmean remained unchanged. A significant decrease in Dmean was observed in the atria. In contrast, no change was observed in the left ventricle, regardless of lymphatic irradiation, due to its anatomical location. These findings suggest that DIBH may be particularly beneficial for patients undergoing IMN irradiation.

The development of CAD is a complex process influenced by multiple risk factors, including age, smoking, hypertension, hypercholesterolemia, and diabetes. These confounding factors complicate establishing a clear dose‐response relationship between radiation dose to the coronary arteries and the development of CAD. Due to its anatomical proximity to the chest wall, the LAD is notably exposed during left breast RT.^28^ In a study by Correa et al., increased cardiac stress markers were observed in patients who underwent left breast RT over a median follow‐up of 12 years, with most complications linked to the LAD. Additionally, 62% of patients who underwent cardiac catheterization had stenosis exclusively in the LAD.^29^ However, another study found no significant difference in coronary angiograms.^30^

The role of RCA dose in breast RT has been investigated less extensively than that of LAD. RCA doses become significant during right breast RT and irradiation of the IM node.^31^ A limited number of studies have suggested a potential association between radiation dose to the RCA and increased cardiovascular event risk.^32^ In our study, the entire RCA was contoured as an OAR, resulting in a higher mean dose than studies focusing solely on the proximal RCA.^31^ Due to its proximity to IM nodes, the RCA was exposed to high radiation doses in free‐breathing (FB) mode during RNI (Dmean 7.9 Gy, V10 29%). With DIBH, the RCA mean dose decreased to 5.1 Gy, and V10 reduced to 11.4%. The LAD received the lowest dose among OARs, and no dose reductions were observed.

One of the notable previous studies in this field is the work by Pandeli et al.,^24^ which provided a comprehensive analysis of the dosimetric effects of DIBH in right‐sided breast cancer radiotherapy. Their study evaluated multiple OARs, which included RCA, and compared patients with and without regional nodal irradiation (RNI), offering valuable insights. However, our study has a more detailed analysis of RCA and subregions of the heart. Notably, while Pandeli et al. did not find a statistically significant reduction in RCA mean dose in patients receiving internal mammary node (IMN) irradiation, our study demonstrated significant dose reductions in all RCA parameters with DIBH, specifically in the subgroup of patients who received IMN irradiation. These findings underscore the potential benefits of DIBH for cardiac sparing, particularly in cases involving more extensive nodal irradiation.

The hepatotoxic effects of breast irradiation remain unclear. Data derived from studies on liver malignancies suggest that keeping the liver mean dose below 28–32 Gy can prevent radiation‐induced liver disease (RILD).^33^ However, the impact of lower doses on the liver is not well understood. In the right breast RT, anatomical factors may lead to radiation exposure to the liver, although doses generally remain below the threshold for RILD. However, caution is warranted in patients with pre‐existing liver disease or those receiving intensive systemic therapy. Cases of radiation‐induced hepatitis and elevations in liver enzyme levels following post‐mastectomy RT have been reported,^34^, ^35^ though these are rarely of clinical significance. In our study, DIBH significantly reduced liver doses, with observed decreases of up to 80% for Dmean, V5, V10, and V20 when compared to FB. The most pronounced benefit of DIBH was observed in the liver. This improvement was consistent across whole‐breast irradiation and RNI, likely due to diaphragmatic movement during inspiration, moving the liver away from the radiation treatment field.

One of the most critical dose‐limiting toxicities in breast RT is radiation pneumonitis. Key dosimetric predictors for pneumonitis include mean lung dose (MLD), D5, and V20.^36^ While these criteria are generally manageable in whole‐breast irradiation, they become more challenging in RNI patients, where a pneumonitis incidence of 4.1% has been reported.^37^ However, modern RT techniques have significantly reduced lung doses, thereby decreasing pneumonitis risk.^38^ Another long‐term concern is secondary lung cancer, with meta‐analytic data suggesting an approximately 11% increased risk per Gy of MLD.^39^ Reducing lung doses is therefore crucial. Given the increased lung volume during deep inspiration, significant improvements in lung dosimetry–‐particularly MLD–‐are expected. Our study also demonstrated dose reductions across all lung dosimetric parameters with DIBH compared to FB, though the effect was less pronounced than in the liver. Notably, the displacement of lung bases from the treatment field may help reduce secondary malignancy risk. Dose reductions were observed in both whole‐breast irradiation and RNI, with the most significant reductions occurring in the RNI group.

The DIBH technique increases treatment costs due to the need for additional CT scans, specialized equipment, and prolonged treatment time. However, the reduction in side effect risks justifies these costs. In left breast RT, cost‐effectiveness studies have shown clear benefits, particularly for patients with underlying heart disease.^40^ However, such studies are lacking for the right breast RT. Our analysis indicated that DIBH prolonged treatment time by approximately 16%, potentially reducing the number of patients treated daily. Due to the limited follow‐up time, we could not perform a comprehensive cost analysis. Nevertheless, based on studies related to left breast cancer, it is likely that DIBH will prove to be cost‐effective.

It has been reported that patients with RNI, can benefit from advanced techniques such as VMAT, which significantly reduce doses to the lung and liver even without the use of DIBH.^35^, ^38^ With FB VMAT, the liver Dmean can be maintained below 4 Gy.^35^ When DIBH is combined with VMAT, particularly for lung doses, significant additional benefits are achieved. Holt et al. suggest that DIBH combined with VMAT should be considered for RNI in right breast cancer.^16^ In our study, patients were treated primarily with IMRT, without limiting the choice of RT technique, and the effect of the RT technique was not analyzed. However, combining DIBH with VMAT may provide the most effective protection for surrounding organs.

In our study, treatment plans for each patient were created using IMRT, 3DCRT, and VMAT, and the most appropriate plan was selected for each case. FB plans were also performed using the same technique to eliminate discrepancies arising from different planning methods. However, potential differences due to planner‐dependent factors cannot be completely ruled out. Another limitation of our study is that the patient population was relatively young, and we excluded patients who received lymphatic irradiation without IMN irradiation, which may restrict the generalizability of our findings. Additionally, the effect of the RT technique was not evaluated; thus, DIBH benefits may diminish when using VMAT or hybrid plans.

This study is the first to simultaneously evaluate multiple OARs and analyze changes in cardiac subunits with DIBH in right‐sided breast RT. While previous studies, have explored dosimetric differences between FB and DIBH, our study adds new insights by performing detailed cardiac substructure and RCA analysis. Due to the short follow‐up period, late adverse effects were not assessed; however, patient follow‐up is ongoing, and future studies are planned to address this issue.

## 5 CONCLUSION

In conclusion, DIBH significantly reduced liver and lung doses. Notable heart, ventricular, atrial, and RCA dose reductions were observed only in patients receiving IMN irradiation.

Our findings suggest that while DIBH may increase treatment duration, the significant reduction in OAR doses could potentially outweigh this drawback, especially for patients with pre‐existing heart conditions. Despite the slight increase in treatment time, the potential benefits of DIBH justify its application for all patients capable of compliance, regardless of lymphatic irradiation.

## AUTHOR CONTRIBUTIONS

All authors contributed to the material preparation, data collection, and analysis. The first draft of the manuscript was written by Semiz V. and all authors commented on previous versions of the manuscript. All authors read and approved the final manuscript.

## CONFLICT OF INTEREST STATEMENT

The authors declare no conflicts of interest.

## ETHICS STATEMENT

This study was approved by the Ethics Committee of Dokuz Eylul University (No: 2025/9959) and conducted in accordance with the principles of the Declaration of Helsinki.

## Supporting information



Supporting information

## Data Availability

The datasets are available from the corresponding author on reasonable request.
