# Supplementary material for: Comprehensive analysis of the role of deep inspiration breath‐hold in right‐sided breast cancer radiotherapy: A focus on cardiac substructures and right coronary artery
Source: J Appl Clin Med Phys. 2025 Aug 21;26(9):e70216. doi: 10.1002/acm2.70216 (PMC12370404; doi:10.1002/acm2.70216)
Supplement: Supplementary file 1 — Supporting information [file ACM2-26-e70216-s001.docx]

**Supplementary Table 1:** Organ-at-Risk Dose Constraints According to Clinical Protocol in Right-Sided Breast Radiotherapy

| Organ at Risk (OAR) | Dosimetric Parameter | Ideal Constraint | Acceptable Constraint |
| --- | --- | --- | --- |
| Ipsilateral Lung | V20  V20 (with RNI)  Dmean | < 20%  <20%  < 12 Gy | < 30%  <35 %  < 18 Gy |
| Contralateral Lung | Dmean | As low as possible | < 5 Gy |
| Heart | Dmean | < 3 Gy | < 5 Gy |
| RCA | Dmean | As low as possible | < 10 Gy |
| LAD | Dmean | As low as possible | As low as possible |
| Liver | Dmean | As low as possible | As low as possible |
| Spinal Cord | Dmax | < 30 Gy | < 45 Gy |
| Contralateral Breast | Dmean  V5 | As low as possible  As low as possible | < 3 Gy  <10% |
